# Ibogaine: Therapeutic Potential, Cardiac Safety, and Translational Perspectives in the Treatment of Substance Use Disorders—A Scoping Review

**DOI:** 10.3390/molecules31030545

**Published:** 2026-02-04

**Authors:** Monica Patrícia Esperança, Nelson G. M. Gomes, Maria Graça Campos

**Affiliations:** 1 Faculty of Pharmacy, Health Sciences Campus, University of Coimbra, 3000-548 Coimbra, Portugal; 2 REQUIMTE/LAQV, Laboratório de Farmacognosia, Departamento de Química, Faculdade de Farmácia, Universidade do Porto, 4050-313 Porto, Portugal; ngomes@ff.up.pt; 3 Coimbra Chemistry Center, Faculty of Sciences and Technology, University of Coimbra, Rua do Norte, 3004-531 Coimbra, Portugal

**Keywords:** addiction neurobiology, cardiac safety, NMDA modulation, pharmacodynamics, pharmacokinetics, psychedelic-assisted therapy, translational research

## Abstract

Substance Use Disorder (SUD) constitutes a major and persistent global public health burden, accounting for approximately 600,000 deaths annually, largely driven by opioid use. Despite substantial advances in addiction neuroscience, currently approved therapeutic strategies remain limited in efficacy, as they predominantly target isolated neurobiological processes and fail to concurrently address core mechanisms such as glutamatergic hyperactivity, mesolimbic hypodopaminergic, and dysfunction of cortical and executive control networks. This mechanistic fragmentation contributes to persistently high relapse rates and underscores the need for integrative and multitarget therapeutic approaches. Within this context, ibogaine has re-emerged as a clinical candidate due to its distinctive multimodal neuropharmacological profile and its reported capacity to modulate multiple pathways implicated in addictive behaviours. However, the clinical translation of ibogaine remains substantially constrained by fragmented and heterogeneous evidence, the absence of regulatory frameworks in several jurisdictions, limited phytochemical validation and standardization of available formulations, and unresolved concerns regarding cardiac safety. This scoping review critically synthesizes the available preclinical and clinical literature on ibogaine in the treatment of SUD, with particular emphasis on reported effects on withdrawal symptoms and craving, dose–response relationships, and the occurrence of cardiac adverse events. By clarifying the current state of the evidence and delineating key translational constraints, this review defines the conditions under which ibogaine, an indole alkaloid isolated from *Tabernanthe iboga* Baill. (Apocynaceae), may warrant continued investigation. The hypothesis of a neurobiological “reset”, supported by emerging preclinical and clinical data, positions ibogaine as a compound of relevance in addiction research and highlights the need for rigorous pharmacological, toxicological, and regulatory evaluation to inform safer and more standardized clinical pathways.

## 1. Introduction

Substance Use Disorders (SUDs) constitute one of the most severe contemporary public health crises and are expected to remain among the greatest global challenges in mental health and addiction medicine over the coming decades. Between 2019 and 2023, the number of opioid users worldwide increased from 54 to 61.5 million, reflecting a sustained and alarming global expansion in opioid consumption, accounting for approximately 600,000 deaths annually, largely driven by opioid use. Parallel increases have been reported for cocaine use and for amphetamine-type stimulants, with an estimated 30.5 million users globally [1,2]. This trajectory underscores the persistent burden of high-risk psychoactive substance use and the urgent need for more effective therapeutic strategies.

Despite the availability of approved pharmacological interventions, namely opioid substitution therapies such as methadone and buprenorphine, and the opioid antagonist naltrexone, clinical outcomes remain limited. High relapse rates, poor long-term adherence, and frequent treatment discontinuation continue to characterize standard care [3,4,5,6]. These limitations have driven growing interest in alternative approaches capable of addressing the neurobiological complexity and persistent motivational dysregulation underlying addictive disorders [7].

**Figure 1:**
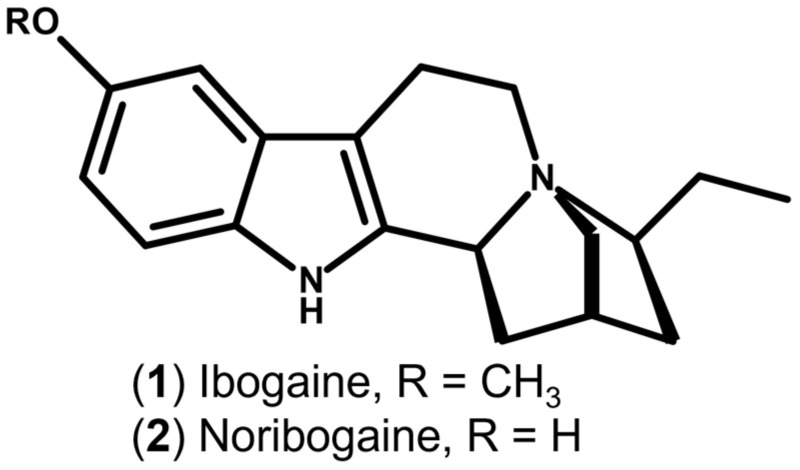
Chemical structures of ibogaine and its primary active metabolite, noribogaine. Ibogaine and noribogaine share a common indole alkaloid scaffold and differ by *O*-demethylation at the methoxy substituent (R=CH_3_ for ibogaine; R=H for noribogaine).

Within this context, *Tabernanthe iboga* Baill., a shrub endemic to Central Africa and traditionally used in Bwiti ritual practices, has gained increasing scientific attention. The root of the plant contains a complex mixture of indole alkaloids, among which ibogaine (Figure 1) is the predominant and most extensively studied compound. Since its chemical characterization in the early twentieth century, and particularly following reports describing rapid attenuation of opioid withdrawal and craving, ibogaine has been progressively investigated as a candidate intervention for SUDs. Preclinical studies in rodent and primate models demonstrate that ibogaine and its active metabolite noribogaine (Figure 1) reduce self-administration of opioids, cocaine, nicotine, and alcohol, supporting its relevance within addiction pharmacotherapy [8,9].

The pharmacological profile of ibogaine distinguishes it from classical psychedelics, such as psilocybin and mescaline, which primarily exert their effects through serotonergic 5-HT2A receptor agonism and do not directly modulate habenular nuclei or striatal dopaminergic signalling [10,11,12,13]. Addiction-related pathology is rooted in dysfunction of mesocorticolimbic and habenular circuits regulating dopaminergic salience, aversion, and motivational control [14]. In contrast to single-target approaches, ibogaine exhibits a distinctive multitarget profile, modulating nicotinic, opioid, glutamatergic and monoaminergic systems, as well as neurotrophic signalling pathways implicated in synaptic plasticity and dopaminergic homeostasis [15,16,17]. Collectively, these actions have been conceptualized as a neurobiological “reset” framework, describing the reconfiguration of addiction-related circuitry accompanied by a window of enhanced plasticity that may facilitate sustained behavioural change [8,18,19]. To the best of current knowledge, no other plant-derived compound has been described as exhibiting a comparable convergence of actions across the MHb–IPN axis, NMDA receptors, monoaminergic systems, neurotrophic signalling, and dopaminergic salience.

Notwithstanding this therapeutic promise, ibogaine is associated with significant safety concerns, including neurotoxic and cardiotoxic effects. Most notably, blockade of the hERG potassium channel and consequent QT interval prolongation imposes a narrow therapeutic window and necessitates rigorous risk-mitigation strategies [20,21,22,23,24]. Importantly, this risk profile must be contextualized within the broader pharmacological landscape, as approved treatments such as methadone, despite their widespread clinical use, are themselves associated with significant relative mortality risks related to QTc prolongation and torsades de pointes [25,26].

**Figure 2:**
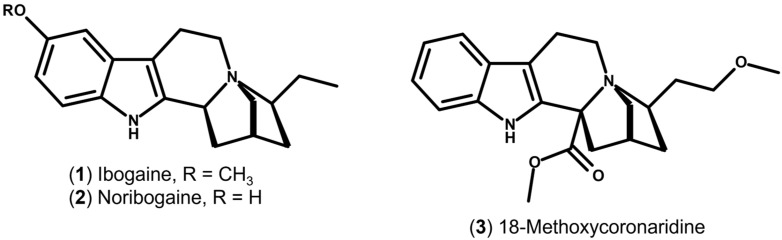
Chemical structures of selected ibogaine analogues. The figure illustrates 18-methoxycoronaridine (18-MC), an ibogaine analogue developed to improve safety profiles while preserving anti-addictive activity.

Structural congeners, including 18-methoxycoronaridine (Figure 2) and oxa-iboga derivatives, have been developed to retain anti-addictive efficacy while exhibiting more favourable cardiac safety profiles in preclinical models [27]. Moreover, variability in ibogaine metabolism to noribogaine, largely driven by cytochrome P450 2D6 (CYP2D6) polymorphisms, contributes to clinically relevant inter-individual differences in exposure and toxicity [28].

Within the European Union (EU), no authorized medicinal products containing ibogaine exist, and any current administration occurs outside regulated pharmaceutical frameworks, without established guarantees of pharmaceutical quality, traceability, or pharmacovigilance. At the international level, the World Health Organization (WHO) has acknowledged the increasing interest in ibogaine as a potential therapeutic agent in the treatment of SUD, while emphasizing the lack of robust clinical data regarding its safety and efficacy, and urging that any research involving psychoactive plant-based substances adhere to rigorous standards of pharmaceutical quality, safety, and ethical oversight [2,29].

The incomplete characterization of ibogaine’s toxicological profile, particularly regarding its cardiotoxic risks, and the absence of harmonized regulatory recognition have resulted in significant international heterogeneity in its legal and clinical status [20,24]. In the EU, no authorized medicinal products containing ibogaine currently exist, nor is *T. iboga* listed in any EMA/HMPC monograph for herbal medicinal use [30], which is to be expected, since it has no traditional use in this territory.

In the absence of recognized traditional use or well-established medicinal use across major regulatory frameworks, ibogaine-containing products, such as extracts of *T. iboga*, are not eligible for simplified authorization pathways and can only be considered within a full medicinal development paradigm, in which duly authorized clinical trials are an indispensable prerequisite. Within this context, references to regulatory quality should not be interpreted as implying regulatory eligibility, but rather as denoting the minimum scientific conditions required to support reproducible, interpretable non-clinical and clinical investigation. Phytochemical standardization may contribute to this objective by mitigating variability inherent to plant-derived materials and can be operationalized through marker-based quantification of ibogaine combined with chromatographic fingerprinting of the alkaloid profile, in accordance with internationally accepted quality-control principles for herbal ingredients [31,32,33].

Taken together, these considerations highlight the need for a rigorous and integrative appraisal capable of reconciling ibogaine’s distinctive pharmacological profile with its safety and regulatory challenges. Accordingly, this scoping review aims to systematically map and critically synthesize the existing clinical, pharmacological, and ethnopharmacological evidence on *T. iboga* and *ibogaine* in the treatment of SUD, with particular emphasis on therapeutic efficacy, cardiac safety, pharmaceutical quality, and translational limitations. By identifying areas of convergence, uncertainty, and evidence gaps, this review seeks to inform future research priorities and support scientifically grounded regulatory and clinical decision-making.

## 2. Results

### 2.1. Overview and Characterization of the Evidence on the Therapeutic Use and Safety of Ibogaine in SUD

The evidence base mapped in this scoping review focuses on ibogaine, the main indole alkaloid of *T. iboga*, in the treatment of SUD, with particular attention to how therapeutic effects and safety risks have been investigated and reported across the literature.

The scientific history of ibogaine spans more than a century, beginning with its isolation in 1901 and the subsequent phytochemical characterization of iboga alkaloids throughout the mid-twentieth century. Although early pharmacological interest remained limited, the seminal observations reported by Lotsof in the 1980s marked a pivotal transition, prompting sustained clinical and mechanistic investigation into ibogaine’s potential therapeutic relevance in SUD, alongside emerging concerns regarding its safety profile.

The body of evidence comprises controlled and observational human studies, as well as pre-clinical and mechanistic research. Across these domains, heterogeneity is evident in study design, exposure forms, dosing approaches, outcome measures, and, most notably, the depth and consistency of safety evaluation, particularly regarding cardiac and neurological effects.

Pre-clinical and experimental studies provide a mechanistic and toxicological context relevant to the interpretation of human findings, while remaining methodologically distinct from clinical research. Collectively, the literature outlines an evidence landscape in which therapeutic exploration and safety concerns have evolved concurrently, supporting the use of a scoping review to map the research domains in which ibogaine has been studied. The following sections present this evidence stratified by study type.

### 2.2. Human Observational Evidence

Across clinical and observational human studies, one of the earliest and most recurrent outcome patterns reported for ibogaine in the treatment of SUD is the acute attenuation of withdrawal symptomatology, occurring shortly after administration and temporally aligned with treatment exposure. Early reports described rapid suppression of withdrawal manifestations after single-dose ibogaine use, providing initial human observations that withdrawal trajectories could be modulated within clinically relevant timeframes [34,35]. These early signals were later extended into clinically monitored investigations, which broadened the scope of reported outcomes beyond acute withdrawal phenomena [36].

Beyond withdrawal-related outcomes, subsequent investigations reported reductions in craving and improvements in mood, extending clinical observations into short- and medium-term neuropsychological domains. Clinically monitored observational studies incorporating standardized outcome measures described significant decreases in craving intensity and mood-related disturbances persisting beyond the immediate post-treatment period [36,37].

Later clinic-based observational studies further reported post-treatment substance use outcomes, including abstinence-related measures and reductions in use across opioid, cocaine, alcohol, and polysubstance use disorders [38,39]. Evidence regarding the duration of post-treatment effects remains heterogeneous. Structured observational follow-up studies conducted within medically supervised protocols reported sustained reductions in substance use extending up to 12 months following treatment in subsets of participants [39,40]. Case-level observations further described remission extending to 18 months at the individual level in treatment-refractory opioid use disorder [41]. Collectively, these findings suggest potential durability of reported effects in selected individuals, alongside substantial interindividual variability in outcome persistence.

Despite decades of observational and naturalistic research on ibogaine for SUD, to date, no randomized, placebo-controlled clinical trials have evaluated its efficacy relative to placebo or established treatments. Accordingly, all available human efficacy data derive from open-label observational studies, retrospective analyses, and case reports, underscoring the need for rigorously designed controlled trials. Currently, ongoing trials are registered, but results remain unavailable.

Clinical and observational evidence on the efficacy of ibogaine in opioid use disorder remains limited, heterogeneous, and methodologically constrained. To date, no randomized controlled trials have been conducted, and the available human evidence consists exclusively of open-label observational studies, retrospective analyses, and case reports. Nevertheless, these studies provide the only empirical basis for evaluating ibogaine’s potential role in opioid detoxification and early post-detoxification outcomes.

In accordance with the scoping review methodology, the present synthesis aimed to map and characterize primary human studies reporting clinical efficacy outcomes of ibogaine in opioid-dependent populations, without attempting to rank evidence or infer comparative effectiveness. Particular attention was given to accurately describing study design, sample size, exposure characteristics, outcome domains assessed, and methodological limitations, to avoid over-interpretation of findings.

Table 1 summarizes the complete body of identifiable primary clinical evidence on ibogaine efficacy in opioid use disorder, spanning from early non-clinical observational case series in the 1990s to more recent medically supervised inpatient detoxification studies. Only studies reporting original human data were included; secondary analyses, narrative reviews, and survey-based self-reports without clinical verification were excluded.

Collectively, the human evidence summarized in Table 1 delineates a consistent but methodologically constrained signal of clinical activity of ibogaine in opioid use disorder. Across heterogeneous observational designs, the most reproducible outcome is the rapid attenuation of opioid withdrawal symptomatology following treatment exposure, reported from early non-clinical case series through to later medically supervised inpatient detoxification studies. This convergence across independent cohorts suggests that ibogaine exerts a robust acute effect on withdrawal-related physiological and subjective domains, temporally aligned with administration and independent of long-term follow-up assumptions.

Beyond acute withdrawal suppression, several studies extended outcome assessment into post-detoxification domains, reporting reductions in craving intensity and improvements in mood during short- and medium-term follow-up. However, these effects were variably sustained, frequently limited to subsets of participants, and strongly influenced by attrition, self-reporting, and incomplete longitudinal verification. Importantly, abstinence-related outcomes were inconsistently defined, heterogeneously measured, and rarely supported by biological confirmation, precluding any reliable estimation of sustained remission rates at the population level.

A critical interpretative advance emerging from later investigations is the recognition that ibogaine’s clinical effects cannot be adequately understood through dose-centric frameworks alone. Studies incorporating pharmacokinetic assessments highlighted substantial interindividual variability driven by CYP2D6-dependent metabolism and prolonged exposure to the active metabolite noribogaine, providing a biologically plausible substrate for the observed heterogeneity in clinical trajectories. This metabolic dimension introduces an additional layer of complexity that limits cross-study comparability and reinforces the need for mechanistically informed interpretation of observational outcomes.

Despite the accumulation of human observational data spanning more than three decades, the current evidence base remains insufficient to establish efficacy in a regulatory or comparative sense. The absence of randomized, placebo-controlled clinical trials, standardized dosing regimens, harmonized outcome measures, and systematic long-term follow-up constitutes a structural limitation that cannot be resolved through further aggregation of naturalistic reports alone. Consequently, the findings summarized herein should be interpreted strictly as descriptive mappings of reported human outcomes rather than confirmatory evidence of therapeutic effectiveness.

Within the framework of a scoping review, these studies nonetheless serve a critical function by defining the empirical boundaries of existing human evidence, identifying recurrent outcome domains, and exposing methodological gaps that must be addressed in future clinical development programs. In this context, the available data support the characterization of ibogaine as a compound with reproducible acute withdrawal-modulating effects and plausible short-term neuropsychological impacts, while simultaneously underscoring the substantial uncertainty surrounding durability, generalizability, and comparative benefit relative to established treatments.

In line with the objectives of a scoping review, the synthesis presented here does not seek to establish efficacy thresholds or clinical recommendations, but rather to delineate the scope, nature, and limitations of the existing human evidence base on ibogaine in opioid use disorder.

### 2.3. Human Safety and Toxicological Evidence

The human safety and toxicological evidence on ibogaine is derived primarily from case reports, forensic investigations, and observational clinical descriptions, with a predominant focus on cardiac electrophysiological disturbances and fatal outcomes temporally associated with exposure. Across these reports, the most consistently described adverse effects involve marked QT interval prolongation and malignant ventricular arrhythmias, reported in both supervised and unsupervised contexts of use [43,44,45,46,47]. The available human safety evidence is inherently incomplete, as ibogaine is frequently used outside regulated medical settings and adverse events may go unreported or lack sufficient clinical documentation.

Multiple case reports document pronounced QTc prolongation following ibogaine administration, in some instances exceeding 600 ms. These electrophysiological disturbances have been observed in hospital settings after therapeutic or quasi-therapeutic use, as well as following ingestion of non-standardized products obtained outside medical control. Reported manifestations include torsades de pointes, ventricular tachycardia, and ventricular fibrillation, occasionally requiring urgent electrical cardioversion or defibrillation [43,44,45,46,47]. Notably, arrhythmic events have been described both after relatively modest reported doses and following very high cumulative exposures, underscoring substantial interindividual variability in cardiac susceptibility [44,45,46,47]. In several cases, QTc prolongation persisted well beyond the acute exposure window, indicating delayed or sustained electrophysiological effects [46].

Fatal outcomes temporally associated with ibogaine exposure have been described in multiple forensic and medico-legal investigations. These include individual fatal case reports as well as aggregated forensic series compiling multiple deaths in which ibogaine exposure was identified as a common factor [20,48,49,50]. Reported fatalities span diverse geographic regions and contexts of use, including alternative treatment settings, unsupervised ingestion, and ritualistic use. Importantly, these cases frequently involved confounding factors, such as pre-existing cardiac or hepatic disease, electrolyte disturbances, polysubstance use (including opioids, methadone, benzodiazepines, or alcohol), and ingestion of non-standardized iboga preparations [20,48,49]. Consequently, although temporal associations between ibogaine exposure and death are consistently reported, causality cannot be unequivocally established based on the available data.

Forensic toxicology studies further contribute to the human safety profile by documenting systemic distribution of ibogaine and its metabolites in post-mortem tissues following acute intoxication or fatal exposure [50,51]. Detection of ibogaine and noribogaine in blood and multiple organs supports the plausibility of sustained systemic exposure, particularly in cases where delayed cardiac events were observed. In some reports, death was ultimately attributed to multifactorial mechanisms, such as drowning or respiratory compromise, with ibogaine exposure being identified as a contributory rather than exclusive factor [48,49].

A recurring theme across both non-fatal and fatal reports is the potential role of pharmacokinetic factors in modulating cardiac risk, particularly the persistence of noribogaine, the primary active metabolite of ibogaine. Case reports incorporating toxicokinetic analyses demonstrate that noribogaine can remain detectable in circulation for extended periods and may contribute to prolonged QTc intervals and delayed arrhythmogenic risk, even after plasma ibogaine concentrations have declined [46]. These observations suggest that cardiac toxicity may not be confined to peak ibogaine exposure but rather reflect combined and temporally dissociated effects of the parent compound and its metabolite, complicating risk prediction based solely on administered dose [44,46].

Collectively, the available human safety and toxicological evidence delineates a consistent pattern of cardiac electrophysiological vulnerability, ranging from reversible QT prolongation to life-threatening arrhythmias and death, reported across heterogeneous contexts of ibogaine use [20,43,44,45,46,47,48,49,50,51]. At the same time, this evidence base is characterized by methodological limitations inherent to case-based and forensic data, including the absence of denominators, incomplete clinical characterization, reporting bias toward severe outcomes, and substantial confounding factors. These features define the current boundaries of human safety knowledge on ibogaine and justify cautious interpretation of reported adverse events within the broader landscape of observational human evidence.

The human safety and toxicological studies underpinning these reported adverse outcome patterns, including context of use, exposure characteristics, and documented cardiac and fatal events, are summarized in Table 2.

The available human safety evidence is inherently incomplete, as ibogaine use often occurs outside regulated medical settings and adverse events may go unreported or lack adequate clinical documentation. The human safety and toxicological evidence on ibogaine is frequently derived from case reports, medico-legal/forensic investigations, and clinical observational descriptions. Across these sources, the most consistently documented adverse outcomes are cardiac electrophysiological disturbances, particularly marked QT/QTc prolongation and malignant ventricular arrhythmias (including torsades de pointes, ventricular tachycardia, ventricular fibrillation, and cardiac arrest), reported after ingestion of mostly non-standardized plant preparations or purportedly purified products. A recurrent feature is the temporal alignment of arrhythmic events with exposure, with QTc values in some reports exceeding 600 ms and with abnormalities persisting beyond the acute exposure window; these observations, while heterogeneous, indicate that clinically relevant risk may extend into a delayed vulnerability phase and is not adequately captured by dose-centric framing alone.

Fatal outcomes temporally associated with ibogaine exposure have been described in individual fatal case reports and aggregated forensic series, but causal attribution is intrinsically constrained by frequent confounding with pre-existing disease, electrolyte disturbances, polysubstance exposure, and non-standardized preparations, and by the absence of denominators and systematic ascertainment. Forensic toxicology studies support the biological plausibility of sustained systemic exposure by demonstrating the detection of ibogaine and noribogaine across multiple post-mortem matrices, consistent with extensive distribution and potential for delayed effects; toxicokinetic case reports further implicate prolonged noribogaine persistence as a determinant of sustained QTc prolongation and delayed arrhythmogenic risk after plasma ibogaine declines. Collectively, within a scoping-review framework, the available human data delineate a coherent pattern of reported cardiac electrophysiological vulnerability associated with ibogaine exposure while remaining methodologically limited by case-based evidence, incomplete documentation, reporting bias toward severe outcomes, and substantial confounding.

### 2.4. Pre-Clinical and Experimental Evidence

Pre-clinical and experimental investigations of ibogaine have been central to defining both its neurotoxic liability and its mechanistic profile, predominantly in rodent models. Across these studies, a consistent dose-dependent pattern of cerebellar vulnerability has been identified, alongside experimental evidence supporting anti-addictive mechanisms at lower or mechanistically distinct exposure ranges.

Experimental studies in rats demonstrated that high doses of ibogaine induce selective cerebellar neurotoxicity, characterized by astrocytosis and degeneration of Purkinje cells. Subsequent dose–response analyses refined this profile, identifying Purkinje cell degeneration at doses ≥50 mg/kg (i.p.) and a no-observed-adverse-effect level (NOAEL) at 25 mg/kg, thereby establishing a quantitative relationship between dose and neurotoxic risk [52,53].

Behavioural investigations further documented dose-related motor disturbances, including tremor and ataxia, reinforcing the functional relevance of cerebellar toxicity and its potential safety implications [54].

In contrast, mechanistic studies addressing ibogaine’s anti-addictive properties demonstrated reductions in drug self-administration mediated by modulation of NMDA receptor activity and κ-opioid signalling, effects that were not directly associated with the high-dose regimens producing cerebellar neurotoxicity [55].

The pre-clinical and experimental studies supporting these findings, including model systems, dose ranges, neurotoxic and mechanistic outcomes, and translational considerations, are presented in Table 3.

The experimental evidence summarized in Table 3 highlights a clear dissociation between ibogaine-induced neurotoxicity and its proposed anti-addictive mechanisms in animal models. Cerebellar neurodegeneration consistently emerges at high doses in rats, particularly following intraperitoneal administration, whereas lower exposure levels or mechanistically distinct paradigms are associated with behavioural and neurochemical effects relevant to substance use disorders.

Importantly, the neurotoxic findings appear to be strongly dose-, species-, and route-dependent. Selective vulnerability of rat cerebellar Purkinje cells has not been uniformly replicated across species, and comparable neuropathological outcomes have not been demonstrated in mice under similar experimental conditions. This species-specific susceptibility underscores the limitations of direct extrapolation of cerebellar toxicity thresholds from rodent models to humans.

Conversely, pre-clinical studies supporting anti-addictive effects implicate a polypharmacological profile involving NMDA receptor modulation, κ-opioid signalling, and alterations in mesolimbic dopamine transmission. These effects are typically observed at exposure ranges distinct from those producing overt neurodegeneration, suggesting that toxic and putative therapeutic mechanisms are not mechanistically inseparable within experimental systems.

Taken together, the pre-clinical literature delineates biological plausibility for both adverse and mechanistically relevant effects of ibogaine, while simultaneously revealing substantial translational uncertainty. Differences in species sensitivity, dosing paradigms, routes of administration, and outcome measures limit the predictive value of animal data for human safety or efficacy. These constraints reinforce the need to interpret pre-clinical findings as boundary-defining rather than directly predictive, providing contextual support—but not confirmation—for observations derived from human studies.

### 2.5. Chemical and Structural Characterization of Ibogaine and Iboga-Type Alkaloids

The chemical investigation of *T. iboga* constitutes one of the earliest examples of systematic phytochemical research applied to a psychoactive medicinal plant. At the beginning of the 20th century, Haller and Heckel conducted pioneering pharmacognostic and chemical studies on *T. iboga* root bark, demonstrating that its principal active constituent was neither a glycoside nor a simple resinous compound, but rather a nitrogen-containing base capable of forming stable salts with acids [56]. This work represents a historical milestone, as it established the alkaloidal nature of ibogaine and laid the foundation for its subsequent structural elucidation and pharmacological investigation.

Ibogaine is chemically classified as a monoterpenoid indole alkaloid, biosynthetically derived from L-tryptophan and belonging to the broader family of iboga-type alkaloids characteristic of several genera within the Apocynaceae. Structurally, ibogaine (12-methoxyibogamine; C_20_H_26_N_2_O) is defined by a rigid polycyclic scaffold comprising an indole moiety fused to a seven-membered tetrahydroazepine ring and a rigid, cage-like bicyclic system characteristic of iboga-type alkaloids, conferring a highly constrained three-dimensional conformation [57,58]. The presence of multiple stereogenic centers, a tertiary amine, and a methoxy substituent at the C-12 position contributes to its pronounced lipophilicity and reinforces its capacity to interact with diverse neurobiological targets.

The structural features outlined above have been mostly elucidated through integrated spectroscopic approaches, in which nuclear magnetic resonance (NMR) plays a central role in the characterization of ibogaine and related iboga-type alkaloids. Extensive NMR datasets for ibogaine, structurally related naturally-occurring alkaloids, and synthetic derivatives are widely available in the literature [59,60]. From a phytochemical perspective, ibogaine does not occur in isolation within *T. iboga*, but rather as part of a chemically diverse alkaloidal matrix. Classical extraction studies and modern analytical investigations have identified several structurally related congeners, including ibogamine, ibogaline, noribogaine, voacangine, tabernanthine, and coronaridine, often present in variable proportions depending on plant origin and extraction method [61,62,63,64]. A concise overview of representative plant sources and extraction approaches reported for ibogaine, and related iboga-type alkaloids is provided in Table 4.

This compositional heterogeneity is relevant, as subtle structural variations within the iboga alkaloid family have been associated with differences in neuroactivity, toxicity, and pharmacokinetic behaviour, reinforcing the need to distinguish between the effects of crude extracts and purified compounds.

Advanced metabolomic profiling by Bading-Taika et al., using LC-MS/MS coupled with database-assisted annotation, significantly expanded the known phytochemical landscape of *T. iboga* root bark. In this study, ibogaine was confirmed as the predominant alkaloid (1.93% *w*/*w*; 24.6% of total chromatographic area), alongside numerous minor indole alkaloids, including noribogaine, coronaridine, tabernantine, and dihydrocoronaridine. In addition, several phenolic constituents, notably caffeoylquinic acids and related derivatives, were identified, suggesting that the bioactivity of *T. iboga* arises from a multifaceted chemical matrix in which neuroactive alkaloids coexist with compounds possessing antioxidant and anti-inflammatory potential [67].

**Figure 3:**
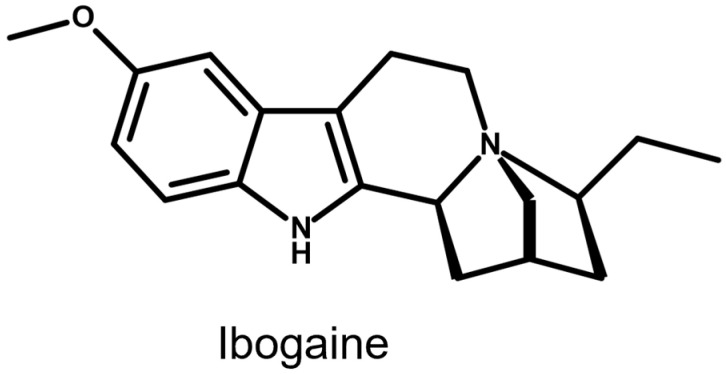
Chemical structure of ibogaine. The structure is shown to illustrate the indole alkaloid scaffold and key functional groups relevant to its chemical characterization.

For illustrative purposes, the chemical structure of ibogaine is shown in Figure 3 to support the chemical description and discussion of its indole alkaloid scaffold and functional groups.

The elucidation of structure–activity relationships within the iboga alkaloid class has directly informed the rational design of second-generation analogues aimed at preserving beneficial bioactivity while reducing toxicity. Early efforts led to the development of 18-methoxycoronaridine (18-MC), a semi-synthetic derivative retaining the iboga scaffold but exhibiting reduced hallucinogenic and cardiotoxic liabilities [58,68].

Despite substantial advances in the rational design of ibogaine-inspired analogues, the translational relevance of these compounds warrants careful and critical evaluation. Drawing on the mechanistic and preclinical evidence summarized above, second-generation analogues such as 18-methoxycoronaridine and oxa-iboga derivatives were developed to preserve anti-addictive efficacy while selectively attenuating liabilities intrinsic to the parent alkaloid. At the mechanistic level, these compounds exhibit a narrower and more target-restricted pharmacological profile, characterized by reduced serotonergic activation and diminished engagement of receptor systems involved in autonomic and cardiac regulation, including sigma and muscarinic acetylcholine receptors [24]. This pharmacological refinement provides a plausible basis for the lower hallucinogenic burden and the attenuation of centrally mediated bradycardic effects consistently observed across preclinical models [55]. Nevertheless, whether such receptor-level selectivity confers a substantively improved cardiac safety margin in humans remains to elucidate. Although current evidence argues against clinically meaningful inhibition of the predominant cardiac sodium channel Nav1.5 at therapeutically relevant concentrations [24], the potential contribution of non-cardiac sodium channel isoforms expressed in human cardiomyocytes, together with the integrated influence of autonomic, electrophysiological, and metabolic determinants, cannot be excluded. Accordingly, while analogue development has demonstrably mitigated specific dose-limiting and centrally mediated toxicities, the available evidence does not substantiate a definitive elimination of cardiotoxic or systemic risk. Rather, the preclinical literature supports a redistribution of pharmacological risk across targets and tissues, underscoring persistent uncertainty regarding human translational safety and highlighting the need for rigorous mechanistic–clinical integration before claims of superior cardiac tolerability can be credibly advanced.

**Figure 4:**
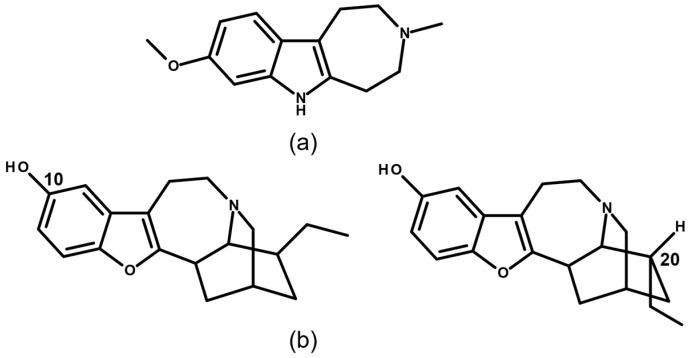
Chemical structures of tabernanthalog (TBG) (**a**) and a representative oxa-iboga derivative (**b**), illustrating structural diversification of the iboga alkaloid scaffold in second-generation analogues. Oxa-iboga analogs are characterized by replacement of the indole moiety with a benzofuran scaffold, a structural modification that reshapes the iboga pharmacological framework and results in enhanced κ-opioid receptor (KOR) engagement. Carbon numbering (C-10 and C-20) highlights key positions commonly targeted for functionalization in iboga alkaloid derivatives.

More recently, function-oriented approaches culminated in the synthesis of tabernanthalog (TBG), a simplified, water-soluble, non-hallucinogenic analogue engineered to capture the psychoplastogenic and anti-addictive pharmacophore of ibogaine while markedly improving safety and drug-like properties [69,70]. In parallel, structurally re-engineered oxa-iboga derivatives have demonstrated potent efficacy in opioid-related preclinical models with substantially attenuated cardiac risk, underscoring the translational potential of iboga-inspired chemotypes beyond the parent natural product [27,71]. Representative chemical structures of these second-generation analogues are shown in Figure 4.

### 2.6. Pharmacodynamic Mechanisms of Ibogaine

The therapeutic relevance of ibogaine in the context of SUD derives from a distinctly polypharmacological profile that enables coordinated modulation of multiple neurobiological systems implicated in addiction. Unlike monotarget pharmacotherapies, such as μ-opioid receptor agonists or selective serotonin reuptake inhibitors, ibogaine engages a convergent network of receptors, transporters, intracellular signalling pathways, and neurotrophic mechanisms [8,16,17,36,72]. This breadth of action is particularly relevant given that SUD does not arise from a single neurochemical perturbation, but from persistent, maladaptive neuroadaptations across reward, aversion, and stress-related circuits [73,74]. From a pharmacodynamic standpoint, ibogaine and its primary active metabolite, noribogaine, interact with a wide spectrum of molecular targets. These include modulation of mesolimbic dopaminergic transmission, interference with the habenular–interpeduncular aversion pathway, partial agonism at opioid receptors, moderate antagonism of NMDA receptors, and the induction of neurotrophic signalling cascades, most notably involving glial cell line-derived neurotrophic factor (GDNF) and brain-derived neurotrophic factor (BDNF). Rather than acting through acute substitution or single-pathway blockade, ibogaine appears to exert its antiaddictive effects through functional recalibration of dysregulated neural circuits.

At the core of ibogaine’s antiaddictive profile lies its capacity to recalibrate mesolimbic dopaminergic signalling, a circuit centrally involved in motivation, reinforcement, and incentive salience. The mesolimbic pathway, comprising dopaminergic neurons projecting from the ventral tegmental area (VTA) to the nucleus accumbens (NAc) and prefrontal cortex (PFC), constitutes the principal substrate through which addictive substances exert reinforcing effects [14,75]. Chronic exposure to drugs of abuse induces supraphysiological dopaminergic stimulation followed by compensatory adaptations, most consistently reflected in downregulation of dopamine D2 receptors in the ventral striatum [76]. This hypodopaminergic state contributes to anhedonia, diminished responsiveness to natural rewards, and compulsive drug seeking. Although both cocaine and ibogaine interact with the dopamine transporter (DAT), their pharmacodynamic consequences differ fundamentally. Cocaine blocks DAT with high affinity, producing abrupt elevations in extracellular dopamine and reinforcing addictive behaviour [36,42]. In contrast, ibogaine inhibits DAT more moderately (Ki ≈ 2 µM), consistent with a modulatory influence on dopaminergic tone during withdrawal states [57]. Importantly, ibogaine also induces expression of GDNF within the VTA, the principal origin of mesolimbic dopaminergic neurons [17]. Preclinical evidence suggests that GDNF signalling supports neuronal survival, synaptic stability, and normalization of dopaminergic transmission, and has been consistently associated with reduced drug self-administration across substance classes [17,69]. While observational human data remain limited and clinical validation is still lacking, these findings support a corrective, rather than stimulatory, role for ibogaine within the reward circuitry.

Addiction is not solely driven by dysregulated reward processing, but also by maladaptive engagement of aversion-related systems, particularly the medial habenula–interpeduncular nucleus (MHb–IPN) axis. This circuit integrates negative motivational signals and exerts inhibitory control over dopaminergic output through projections to the VTA and raphe nuclei [77,78]. Chronic substance exposure induces compensatory hyperactivation of this pathway, resulting in persistent dysphoria and anxiety during abstinence, key drivers of negative reinforcement and relapse. Ibogaine and its synthetic analogue 18-methoxycoronaridine (18-MC) exert functional antagonism at nicotinic acetylcholine receptors containing α3β4 subunits, which are highly expressed in the medial habenula. In preclinical models, blockade of α3β4-containing receptors attenuates MHb–IPN hyperactivity and is associated with marked reductions in self-administration of opioids, stimulants, alcohol, and nicotine [57,79,80]. Although clinical validation in humans remains limited, the consistency of these findings across substances supports this pathway as a distinctive and potentially translational target of ibogaine’s antiaddictive action.

Noribogaine further contributes to ibogaine’s pharmacodynamic profile through sustained modulation of the endogenous opioid and monoaminergic systems. Acting at μ- and κ-opioid receptors, noribogaine contributes to the attenuation of withdrawal-related symptoms without inducing the pronounced euphoria characteristic of full μ-opioid agonists [60]. This profile enables mitigation of both somatic and affective components of withdrawal, including dysphoria mediated by κ-opioid receptor signalling [81], while minimizing the risk of substitution dependence. In parallel, noribogaine has been shown to modulate serotonergic neurotransmission, consistent with reduced serotonin reuptake and enhanced serotonergic signalling within corticolimbic circuits arising from the raphe nuclei [9,60]. This serotonergic modulation contributes to anxiolytic and antidepressant-like effects during the post-acute withdrawal phase. This metabolic dependency reinforces the notion that ibogaine’s pharmacodynamics cannot be dissociated from its pharmacokinetic variability, as CYP2D6-dependent conversion to noribogaine governs both persistence of therapeutic effects and interindividual cardiovascular risk, including QT interval prolongation [28,82].

Complementing these mechanisms, ibogaine exhibits noncompetitive antagonism at NMDA-type glutamate receptors (IC_50_ ≈ 1–2 µM). While early hypotheses emphasized NMDA antagonism as a central antiaddictive mechanism [83], subsequent evidence suggests that this pathway is neither necessary nor sufficient to account for ibogaine’s core effects but may contribute contextually to reduced excitotoxicity and attenuation of drug-associated memory reinstatement during withdrawal [8,84]. Crucially, these acute neurochemical effects are embedded within a broader framework of activity-dependent neuroplasticity. Ibogaine and noribogaine induce durable adaptations through increased expression of neurotrophic factors, most consistently GDNF and, context-dependently, BDNF, in key reward-related regions such as the VTA and NAc [17,69]. At the intracellular level, activation of mTOR-dependent pathways supports synaptic remodelling and corrective reorganization of maladaptive circuitry [12,85].

Collectively, these findings support a model in which ibogaine acts not through acute substitution or receptor dominance, but through coordinated recalibration of reward, aversion, and plasticity networks. This deliberately reiterated polypharmacological profile distinguishes ibogaine from monotarget addiction pharmacotherapies and underpins its unique, though still incompletely validated, therapeutic potential. From a pharmacodynamic perspective, ibogaine represents one of the most comprehensive attempts to pharmacologically address the system-level nature of addiction, while simultaneously underscoring the need for rigorous translational evaluation considering remaining safety and clinical-validation constraints.

### 2.7. Mechanistic Basis of Ibogaine-Associated Cardiotoxicity and Prolonged Risk Window

While the polypharmacological architecture of ibogaine provides a compelling mechanistic rationale for its proposed antiaddictive effects, the same systems-level engagement that supports its therapeutic plausibility also gives rise to clinically relevant safety liabilities. In particular, the interaction of ibogaine and its active metabolite with cardiac ion channels, together with their complex and highly variable pharmacokinetic disposition, introduces a substantiated risk of delayed and potentially severe cardiotoxicity. Addressing this risk requires a mechanistic analysis that extends beyond clinical outcomes to the molecular, electrophysiological, and exposure-dependent processes governing cardiac excitability and temporal vulnerability.

A converging body of mechanistic and pharmacokinetic evidence indicates that the principal safety limitation of ibogaine therapy in SUD lies in its propensity to induce cardiac electrical instability through concentration-dependent prolongation of ventricular repolarization. At the cellular level, ibogaine and its active metabolite noribogaine interfere with cardiac potassium currents involved in phase 3 repolarization, most notably the rapid delayed rectifier current (IKr) mediated by the human Ether-à-go-go–Related Gene (hERG) channel. Inhibition of hERG/IKr reduces repolarization reserve, facilitating early afterdepolarizations and creating a well-established substrate for QT/QTc interval prolongation with potential progression to torsades de pointes and malignant ventricular arrhythmias [24].

Beyond parent-compound effects, a key determinant of cardiac risk is ibogaine’s complex disposition and its biotransformation to noribogaine, with marked interindividual variability driven largely by 2D6. Controlled human pharmacokinetic studies demonstrated that reduced CYP2D6 activity substantially increases systemic exposure to the combined active moiety (ibogaine plus noribogaine), thereby amplifying both the magnitude and duration of electrophysiological liability [28]. Consistent with this mechanism, a randomized, double-blind, placebo-controlled ascending-dose study established a clear concentration-dependent increase in QTc associated with noribogaine exposure, directly linking circulating metabolite levels to delayed ventricular repolarization in humans [82].

The clinical relevance of this exposure-driven risk is further reinforced by toxicokinetic observations demonstrating sustained QTc prolongation and ventricular arrhythmias persisting for several days after ibogaine ingestion, despite near-complete clearance of the parent compound from plasma, implicating noribogaine as a principal driver of prolonged electrical instability [46].

In addition to metabolic determinants, tissue distribution represents a complementary mechanistic contributor to delayed toxicity. Ibogaine exhibits marked lipophilicity and preferential sequestration in adipose tissue, where concentrations exceed plasma levels by more than two orders of magnitude shortly after administration in animal models. This adipose compartment functions as a peripheral pharmacological reservoir, enabling slow redistribution of ibogaine back into the systemic circulation and thereby sustaining low-level exposure to the parent compound beyond the acute dosing phase. When considered alongside the slow elimination kinetics of noribogaine and its concentration–QTc relationship, this distributional property provides a mechanistically coherent explanation for the prolonged arrhythmic risk window described in humans, in which cardiac electrical instability may emerge or persist days after administration despite apparent resolution of acute exposure [28,82,86,87].

Taken together, these findings delineate an integrated mechanistic framework in which hERG/IKr inhibition establishes the electrophysiological substrate for QT/QTc prolongation, while CYP2D6-dependent metabolism, slow elimination of noribogaine, and lipophilic tissue sequestration synergistically extend the temporal window of cardiac vulnerability [24,28,82,87]. This framework provides a robust explanation for delayed, severe, and potentially fatal arrhythmic events reported after ibogaine exposure.

## 3. Discussion

The evidence mapped in this scoping review supports the view that ibogaine occupies a singular pharmacological position among compounds investigated for SUDs, combining broad antiaddictive activity with a mechanistic architecture that is both therapeutically compelling and intrinsically constraining. Across preclinical and observational studies, interpreted in light of complementary mechanistic evidence, ibogaine consistently emerges as a compound whose effects cannot be reduced to a single molecular target or neurotransmitter system, but instead reflect convergent neurochemical and circuit-level actions.

From a neurobiological perspective, ibogaine’s antiaddictive profile is consistent with simultaneous engagement of dopaminergic, serotonergic, opioid, glutamatergic, and nicotinic systems. Modulation of monoamine transporters, including DAT and SERT, contributes to acute alterations in extracellular neurotransmitter levels within mesolimbic and striatal regions. However, the heterogeneity observed across ibogaine, noribogaine, and 18-methoxycoronaridine (18-MC) indicates that monoamine reuptake inhibition alone does not account for sustained reductions in drug-seeking behaviour [55,88]. Notably, the dissociation between serotonergic activation and antiaddictive efficacy, most clearly reflected in the absence of serotonin release following 18-MC administration, argues against serotonergic mechanisms as necessary drivers of therapeutic effects, while positioning them more plausibly within the hallucinogenic and psychotropic dimensions of ibogaine exposure [88].

More convergent evidence supports the involvement of the medial habenula–interpeduncular nucleus axis and α3β4 nicotinic acetylcholine receptors as a critical substrate for antiaddictive actions. This circuitry, centrally implicated in aversion, negative reinforcement, and withdrawal-related dysphoria, provides a mechanistic framework capable of integrating reductions in self-administration across opioids, stimulants, nicotine, and alcohol [55,58]. Importantly, this habenular–nicotinic pathway is retained, and in some analogues potentially sharpened, supporting its relevance as a core antiaddictive mechanism rather than a compound-specific epiphenomenon.

Beyond acute neurotransmission, the mapped literature emphasizes the persistence of ibogaine-associated behavioural effects well beyond the period of direct receptor occupancy. Induction of neurotrophic signalling, particularly involving glial cell line–derived neurotrophic factor (GDNF) within the ventral tegmental area, has been repeatedly associated with longer-lasting reductions in drug seeking and relapse vulnerability [55,89]. This neuroadaptive dimension aligns ibogaine with an emerging class of psychoplastogenic compounds capable of driving durable structural and functional remodelling of circuits implicated in motivation and reward [70]. Within this interpretative framework, ibogaine’s therapeutic relevance extends beyond transient suppression of craving or withdrawal and is more coherently understood as engaging mechanisms compatible with circuit-level recalibration.

It should be noted that the present discussion reflects a narrative integration of heterogeneous preclinical and observational evidence, rather than a formal comparison of effect sizes or causal inference. As such, mechanistic interpretations are presented to contextualize observed therapeutic and safety signals, rather than to establish definitive pathways of action.

However, the same pharmacological breadth that underpins ibogaine’s plausibility also constrains its translational viability. Mechanistic and electrophysiological evidence indicate that inhibition of the hERG/IKr potassium channel constitutes a primary liability intrinsic to the ibogaine scaffold [24]. This risk is further compounded by CYP2D6-dependent formation of noribogaine, slow metabolite elimination, and extensive lipophilic tissue sequestration, collectively producing a prolonged window of cardiac vulnerability that is poorly aligned with acceptable safety margins for clinical deployment [28,46,82,87]. Importantly, convergent pharmacokinetic–pharmacodynamic relationships link circulating noribogaine concentrations to QTc prolongation in humans, reinforcing the clinical relevance of this liability.

Parallel constraints emerge from preclinical evidence of dose-dependent cerebellar neurotoxicity, including Purkinje cell degeneration at high exposures [60]. While translational extrapolation from rodent models requires caution, the reproducibility and specificity of this signal support the conclusion that ibogaine’s therapeutic window is constrained not by isolated adverse effects, but by structural features that couple efficacy and toxicity within overlapping exposure ranges [52].

Collectively, these observations support reframing ibogaine not as a viable therapeutic endpoint, but as a lead compound that defines a pharmacological space of substantial interest. The development of 18-MC illustrates that selective targeting of α3β4 nicotinic receptors can preserve antiaddictive activity while attenuating tremorigenicity, cerebellar toxicity, and hERG affinity [24,55,68]. At the same time, the reduced engagement of neurotrophic pathways by 18-MC highlights a central design challenge: improving safety without forfeiting plasticity-related benefits that may contribute to durability of response.

Recent advances in function-oriented synthesis further reinforce this trajectory. Simplified ibogaine-inspired scaffolds, including TBG and related analogues, retain psychoplastogenic properties while exhibiting reduced hERG liability and improved physicochemical profiles [57,70].

## 4. Materials and Methods

### 4.1. Study Design

This work was conducted as a scoping review, an approach particularly suited given the complex pharmacological profile of ibogaine, the diversity of experimental models and exposure contexts reported in the literature, and the absence of randomized controlled clinical trials in humans.

The scoping review framework was selected to comprehensively capture empirical evidence on therapeutic efficacy and safety outcomes associated with ibogaine and/or noribogaine in the context of SUDs, while also identifying translational limitations and gaps in the existing literature. The review was conducted in accordance with the Preferred Reporting Items for Systematic Reviews and Meta-Analyses extension for Scoping Reviews (PRISMA-ScR).

In addition to the core evidence mapping, this work incorporated a structured mechanistic and chemical synthesis, drawing on foundational pharmacological, electrophysiological, and structural studies. These sources were used exclusively to contextualize and interpret the mapped human and pre-clinical empirical evidence and were not considered part of the scoping review evidence corpus or the PRISMA-ScR quantitative reporting.

### 4.2. Search Strategy

A comprehensive and systematic literature search was conducted primarily using PubMed/MEDLINE, SciELO, and ScienceDirect. The Cochrane Library and the Regional Portal of the Virtual Health Library (BVS) were additionally consulted to identify complementary records. Google Scholar was selectively used as a supplementary source to retrieve relevant publications not indexed in the primary databases. For PRISMA-ScR reporting purposes, all retrieved records, regardless of the platform used for full-text access, were attributed to their original indexing database and counted only once in the study selection process.

The temporal scope of the search extended from 1901 to 2025. This broad timeframe was intentionally defined to capture the historical trajectory of ibogaine research, from its initial isolation and early pharmacognostic and chemical characterization to contemporary investigations addressing therapeutic outcomes and safety liabilities.

Search terms were applied to titles, abstracts, and keywords and included combinations of “ibogaine”, “ibogaine AND substance use disorder”, “ibogaine AND addiction”, and “ibogaine AND withdrawal”, as well as mechanism- and disposition-related terms (e.g., “pharmacodynamics”, “pharmacokinetics”, “NMDA”, “glutamate”, “metabolism”, and “CYP2D6”). To ensure systematic coverage of safety-related evidence, additional terms addressing toxicity and adverse outcomes (e.g., cardiotoxicity, QT/QTc prolongation, arrhythmia, neurotoxicity, and noribogaine) were incorporated. Given the frequent contextualization of ibogaine within psychedelic-assisted frameworks, supplementary searches using broader terms (e.g., “psychedelic-assisted therapy” and “psychedelics”) were conducted where appropriate. Reference lists of eligible publications were manually screened to identify additional relevant studies not retrieved through database searches, particularly older or cross-disciplinary articles relevant to translational research.

Early foundational works, including laboratory-based botanical and chemical characterizations of *Tabernanthe iboga*, were considered essential to contextualize subsequent neuropharmacological and toxicological findings. The search strategy acknowledged the structural constraints of the field, notably the predominance of preclinical, observational, case-based, and post-mortem studies, as well as the absence of randomized controlled trials in humans.

In parallel with the systematic database search, targeted searches were conducted on publisher platforms and academic search engines to retrieve full texts of identified studies and to locate foundational mechanistic, electrophysiological, chemical, and analogue-focused publications used exclusively for contextual interpretation. These targeted searches were not intended to expand the systematically mapped evidence corpus and were therefore excluded from quantitative reporting under PRISMA-ScR.

### 4.3. Eligibility Criteria

Studies were considered eligible for inclusion if they investigated ibogaine and/or its primary active metabolite, noribogaine, in relation to therapeutic effects, safety outcomes, or toxicological profiles. The scoping review encompassed human observational and naturalistic studies, case series, forensic and toxicological reports, as well as pre-clinical experimental investigations in animal models, providing empirical data relevant to the interpretation of human findings. Only publications written in, or reliably translated into, English, Portuguese, or Spanish and published in peer-reviewed scientific journals were included.

Eligibility criteria were applied in a domain-specific manner, reflecting the distinct scientific objectives of the therapeutic and safety evidence streams. For the assessment of therapeutic efficacy, inclusion was restricted to studies involving individuals diagnosed with SUDs, as defined by the respective authors, and reporting clinically relevant outcomes such as withdrawal attenuation, craving reduction, abstinence, or substance use trajectories. In contrast, human safety and toxicological evidence were intentionally evaluated independently of clinical indication. Studies reporting cardiac, neurological, or fatal adverse events following ibogaine exposure were included regardless of whether exposure occurred in the context of SUD treatment, alternative therapeutic settings, ritual use, or unsupervised ingestion, as these adverse effects reflect intrinsic exposure-related properties of ibogaine rather than disease-specific factors.

Pre-clinical animal studies were included when they provided empirical toxicological or pharmacological outcome data relevant to human safety interpretation, including dose-dependent toxicity, behavioural effects, or physiological outcomes. These studies were not required to model SUDs explicitly, as their primary relevance lay in informing biological plausibility, safety boundaries, and risk characterization.

Studies were excluded if they did not involve ibogaine or noribogaine, addressed outcomes unrelated to therapeutic effects or safety, lacked peer review, or provided insufficient methodological detail to allow assessment of relevance. Articles without an accessible abstract were excluded. For the therapeutic efficacy domain, isolated case reports involving severe psychiatric disorders or major medical comorbidities unrelated to SUDs were excluded to minimize confounding; these exclusions were not applied to safety and toxicological analyses.

Eligibility criteria were therefore explicitly domain-specific by design. While therapeutic efficacy studies were restricted to populations diagnosed with SUDs, safety and toxicological evidence were deliberately assessed independently of clinical indication, reflecting the exposure-dependent nature of ibogaine-associated risks.

### 4.4. Study Selection

The study selection process followed a structured, multi-stage screening procedure consistent with PRISMA-ScR recommendations and tailored to the multidisciplinary scope of ibogaine research. A total of 199 records were identified through database searching, including PubMed/MEDLINE (*n* = 152), SciELO (*n* = 31), and the Cochrane Library (*n* = 16). After inter-database deduplication, 38 duplicate records were removed, resulting in 161 unique records eligible for title and abstract screening. Although full-text articles were retrieved from multiple publisher platforms, including ScienceDirect (Elsevier) and others, records were attributed to databases based on their original identification source for PRISMA reporting purposes.

Title and abstract screening assessed relevance to the predefined scope, focusing on studies that investigated ibogaine and/or noribogaine and addressed therapeutic efficacy, safety, or toxicological outcomes. During this phase, 112 records were excluded because they did not investigate ibogaine or noribogaine, did not address therapeutic effects or safety outcomes, focused exclusively on ethnobotanical, sociocultural, or historical aspects, or were outside the predefined scope of the scoping review. This resulted in 49 full-text articles assessed for eligibility.

Full-text eligibility assessment was subsequently conducted using the predefined inclusion and exclusion criteria. Of the 49 full-text reports evaluated, 27 were excluded with explicit reasons, including absence of original empirical data (e.g., reviews, perspectives, editorials), protocol descriptions without reported outcomes, insufficient data on therapeutic efficacy or safety, and overlapping or duplicate study populations.

Following this final eligibility assessment, 22 studies met all inclusion criteria and were retained for qualitative synthesis. The included studies were grouped into three empirical domains: (i) observational human studies evaluating therapeutic efficacy in SUDs; (ii) human safety and toxicological evidence, including case reports and observational data independent of clinical indication; and (iii) pre-clinical animal studies providing empirical evidence relevant to ibogaine’s pharmacological and toxicological profile, as well as safety interpretation. Mechanistic, chemical, and analogue-focused studies were used exclusively for contextual and interpretative purposes in dedicated background sections and were not included in the scoping review evidence corpus.

**Figure 5:**
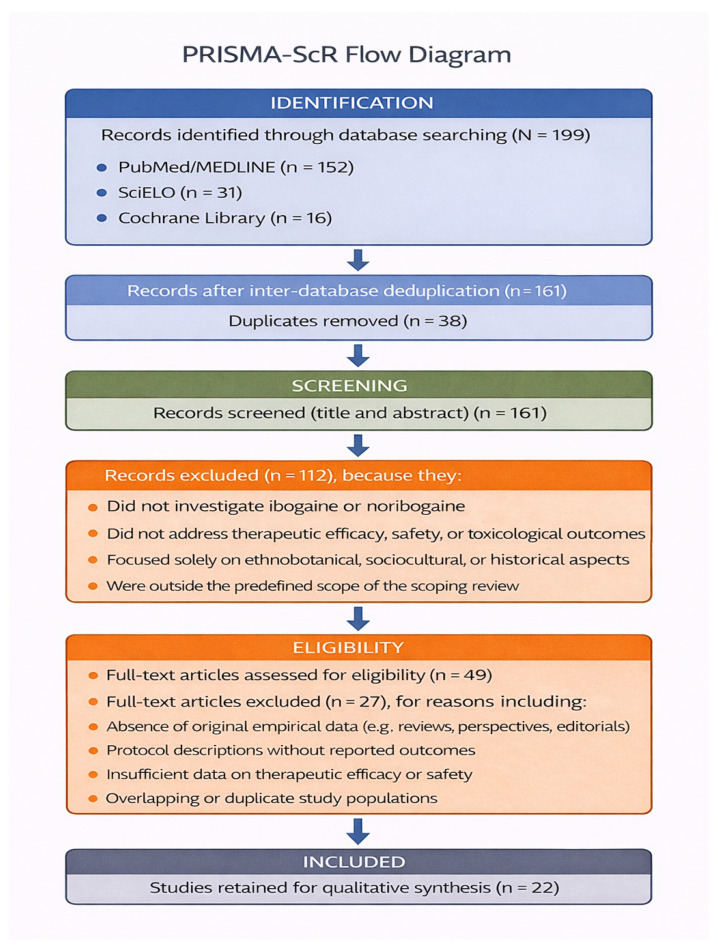
PRISMA-ScR flow diagram illustrating the study selection process (identification, screening, eligibility assessment, and final inclusion) based on predefined eligibility criteria.

Study selection was performed independently by three reviewers. Discrepancies were resolved through discussion and cross-validation, ensuring consistency and methodological rigor. The complete study selection process, encompassing identification, screening, eligibility assessment, and final inclusion, is summarized in the PRISMA-ScR flow diagram (Figure 5).

### 4.5. Data Extraction

Data extraction was performed using a standardized data-charting form developed in a structured matrix format. For each included study, information was systematically collected on study design and setting, population characteristics or experimental model (human, animal, or in vitro), and the compound investigated, including ibogaine and/or noribogaine as the primary compounds of interest, as well as structurally related analogues (e.g., 18-methoxycoronaridine) when evaluated in pre-clinical models. Exposure characteristics, including dose and dosing regimen, were recorded when reported. Key outcome variables were extracted in accordance with the predefined scoping domains and included therapeutic efficacy outcomes (e.g., withdrawal attenuation, craving reduction, abstinence, and substance use trajectories) and safety/toxicological outcomes, with particular emphasis on cardiac and neurological events, including fatal outcomes when reported.

### 4.6. Data Synthesis

The synthesis of findings was descriptive and narrative in nature. Given the substantial heterogeneity of study designs, experimental models, exposure contexts, and outcome measures, no quantitative synthesis or meta-analysis was performed. Instead, results were integrated conceptually to identify convergent and divergent findings across therapeutic efficacy, safety, and pre-clinical evidence streams, and to highlight translational limitations between pre-clinical observations and human outcomes.

### 4.7. Data Availability

All data included in this scoping review are presented within the tables and figures of the manuscript. No external datasets were generated or analysed. Additional information is available from the corresponding author upon reasonable request.

### 4.8. Use of Generative Artificial Intelligence

Generative artificial intelligence tools were used solely to assist with the structural organization of the manuscript, figure preparation, and bibliographic reference management. These tools were not used for study selection, data extraction, data interpretation, or scientific analysis. All scientific decisions and interpretations remain the sole responsibility of the authors.

## 5. Conclusions

Taken together, the findings discussed here indicate that the therapeutic promise of ibogaine lies less in its direct clinical application than in its capacity to inform the rational design of next-generation compounds capable of preserving circuit-level antiaddictive efficacy while meeting contemporary safety and regulatory standards.

Future research should therefore prioritize medicinal chemistry strategies explicitly aimed at uncoupling psychoplastogenic and antiaddictive efficacy from cardiac electrophysiological risk. Early integration of hERG screening, human-relevant cardiomyocyte models, and comprehensive metabolite profiling should be regarded as core components of translational development pipelines.

In parallel, mechanistic studies clarifying the relative contributions of GDNF induction, habenular modulation, and activity-dependent synaptic plasticity to sustained behavioural outcomes will be essential for defining target-engagement benchmarks beyond acute neurotransmitter modulation. Collectively, these directions position ibogaine not as an endpoint, but as a conceptual and structural bridge toward a second generation of anti-addictive therapeutics with improved safety, selectivity, and translational viability.

## Figures and Tables

**Table 1:** Human Studies Evaluating Therapeutic Efficacy of Ibogaine in Substance Use Disorders (SUDs).

Author (Year)	Study Design	N	Exposure/Dosing	Main Efficacy Outcomes	Methodological Limitations
Sheppard (1994) [34]	Open-label observational case series	7	Oral ibogaine HCl (verified purity > 98%) in capsule form (test dose: 100–200 mg; total dose: 700–1800 mg); acute psychoactive phase (≈24–38 h).	Clinically significant opioid withdrawal symptoms were fully suppressed in all participants (0/7) after the acute psychoactive phase. Follow-up showed dose-related variability: the participant who received 700 mg relapsed within 2 days; among those receiving ≥1000 mg (*n* = 6), two relapsed after several weeks, one reverted to intermittent opioid use, and three remained abstinent for ≥14 weeks.	Very small sample size; open-label design; absence of control group; non-standardized outcome assessment; reliance on self-report for follow-up drug use; non-clinical setting.
Alper et al. (1999) [35]	Open-label observational case series (non-clinical setting)	33	Oral ibogaine administration; non-standardized dosing; observation period up to 72 h post-treatment.	Resolution of opioid withdrawal signs within 24 h in 25/33 patients, sustained over 72 h without further drug-seeking behaviour	Open-label design; absence of control group; non-clinical setting; short observation window; non-standardized dosing and formulation
Mash et al. (2000) [36]	Open-label observational clinical study with pharmacokinetic analysis	27	Single oral dose of ibogaine HCl with pharmacokinetic monitoring	Significant reduction in cocaine and heroin craving and depressive symptoms during detoxification and sustained at 30-day follow-up	Open-label, non-randomized design; limited sample size; absence of a control group; self-reported outcomes
Schenberg et al. (2014) [38]	Retrospective observational study	75	Ibogaine is administered under medical supervision and combined with psychotherapy	61% self-reported abstinence at retrospective assessment; statistically significant prolongation of abstinence duration compared with pre-treatment periods (median 5.5 months after single treatment; 8.4 months after multiple treatments; *p* < 0.001)	Retrospective design; reliance on self-reported outcomes; absence of a control group; heterogeneous substance use
Brown & Alper (2018) [6]	Observational study (naturalistic, non-randomized)	30	Mean total dose 1540 ± 920 mg ibogaine HCl (mean ~19 mg/kg); administered in non-medical clinic settings with short inpatient stay (3–6 days)	Marked reduction in opioid withdrawal severity within 72 h (SOWS: −17 points; *p* < 0.001) and significant reductions in opioid use relative to pretreatment baseline, with maximal effect at 1 month and partial maintenance up to 12 months in a subset of subjects.	Observational design; absence of control group; attrition over long-term follow-up; reliance on self-reported drug use; heterogeneity of sustained response.
Noller et al. (2018) [40]	Observational 12-month follow-up study	14 (8 completers at 12 months)	Single ibogaine treatment (25–55 mg/kg ibogaine HCl; staggered dosing over 24–96 h)	Significant acute reduction in opioid withdrawal symptoms (SOWS, *n* = 14); significant reduction in ASI-Lite drug use scores at 12 months among completers (*n* = 8, *p* = 0.002); sustained reduction in depressive symptoms (BDI-II, *p* < 0.001); opioid cessation or sustained reduced use over 12 months in a subset of participants	Small sample size; high attrition (12-month completers *n* = 8); partial reliance on self-report; incomplete biological verification of drug use; absence of a control group; one treatment-related fatality reported
Mash et al. (2018) [42]	Open-label observational case series (inpatient medically supervised detoxification)	191	Oral dose of ibogaine HCl (8–12 mg/kg) administered under medical supervision; pre-treatment opioid stabilization; continuous ECG and laboratory monitoring; pharmacokinetic assessment of ibogaine/noribogaine	Marked reduction in opioid withdrawal severity; significant reduction in opioid and cocaine craving during detoxification and at 1-month follow-up (where available); significant improvement in depressive symptoms; PK data demonstrating ibogaine–noribogaine conversion and prolonged noribogaine exposure	Open-label design; no control group; heterogeneous substance use (opioids and cocaine); short and incomplete follow-up; reliance partly on self-report; outcomes focused on detoxification and short-term transition rather than sustained abstinence
Cloutier-Gill et al. (2016) [41]	Case report	1	Four-day ibogaine treatment (oral administration; exact dose and formulation not reported)	37-year-old female with 19-year history of severe opioid use disorder achieved sustained opioid abstinence at 18-month follow-up after ibogaine treatment; previous longest continuous abstinence was two months while on methadone; no safety issues reported during or after treatment	Single-case design; absence of control or comparator; lack of dosing and pharmacokinetic details; outcomes not generalizable; abstinence outcome based on case description and clinical follow-up rather than systematic or blinded assessment

**Table 2:** Human Studies Reporting Safety, Toxicity, and Adverse Events Associated with Ibogaine.

Author (Year)	Study Type	N	Exposure/Dosing	Main Safety Findings	Methodological Limitations
Hoelen et al. (2009) [43]	Case report	1	Oral exposure to a non-standardized ibogaine preparation (15% ibogaine; total dose 3.5 g), administered in a non-regulated alternative medicine setting	Severe QTc prolongation (QTc up to 616 ms) with documented ventricular tachyarrhythmias temporally associated with ibogaine exposure; QT interval normalized approximately 42 h after cessation despite correction of electrolyte abnormalities, indicating a strong temporal association between ibogaine exposure and malignant cardiac electrophysiological disturbances	Single-case design; absence of standardized or purified formulation; high-dose, imprecisely characterized exposure; concomitant electrolyte disturbances; no pharmacokinetic or genetic assessment; limited causal inference despite strong temporal relationship
Alper et al. (2012) [20]	Systematic retrospective forensic review of fatality cases	19	Ibogaine ingestion in medical and nonmedical settings; heterogeneous preparations and doses, including ethnopharmacological forms; exposure-to-death interval 1.5–76 h; dosing and formulation frequently undocumented or imprecise	19 deaths temporally associated with ibogaine ingestion. No consistent neurotoxic syndrome identified. Postmortem and clinical evidence indicated that preexisting cardiovascular disease and/or concomitant use of other substances explained or contributed to death in most cases, with adequate data. Additional risk factors included alcohol or benzodiazepine withdrawal–related seizures and uninformed use of non-standardized ibogaine preparations.	Retrospective design; reliance on medico-legal reports; heterogeneous and incomplete exposure data; frequent absence of standardized dosing, formulation, pharmacokinetic, or genetic information; multiple confounding factors; temporal association without definitive causal attribution.
Steinberg & Deyell (2018) [44]	Case report	1	Oral ingestion of ibogaine capsules obtained in a non-medical, non-regulated setting; estimated total dose of 65–70 mg/kg (highest survived dose reported); non-standardized preparation with uncertain alkaloid content; no serum ibogaine quantification performed	Extreme QTc prolongation (QTc up to 714 ms) mimicking acquired long-QT syndrome type 2, complicated by ventricular flutter/ventricular tachyarrhythmia at 270 bpm and near-fatal cardiac arrest requiring emergent defibrillation. Delayed QT recovery required approximately 7 days despite electrolyte correction, consistent with prolonged exposure to the active metabolite noribogaine. Strong temporal association between ibogaine ingestion and malignant ventricular arrhythmia.	Single-case design; estimated rather than measured dose; non-standardized ibogaine formulation; absence of serum ibogaine/noribogaine concentrations; presence of secondary hypokalemia as a proarrhythmic facilitating factor; findings not generalizable despite strong mechanistic and temporal coherence.
Pleskovic et al. (2012) [45]	Case report	1	Oral ibogaine exposure (600 mg); non-regulated setting; objective confirmation of ibogaine and noribogaine in blood by LC-MS/MS; no co-ingested substances detected except low-level methadone.	Recurrent malignant ventricular arrhythmias (≥5 episodes of ventricular fibrillation and multiple ventricular tachycardias) associated with marked and persistent QTc prolongation (up to 593 ms), lasting up to 9 days post-exposure; arrhythmic events temporally aligned with ibogaine/noribogaine plasma levels and frequently triggered by vagal maneuvers (micturition/defecation); exclusion of congenital long-QT syndrome and structural heart disease supports drug-induced cardiotoxicity.	Single-case design; non-standardized product; prior opioid and cocaine abstinence; partial contribution of low-level methadone and amiodarone effects cannot be fully excluded; absence of controlled conditions limits generalizability despite strong temporal, toxicological, and electrophysiological evidence.
Mazoyer et al. (2013) [48]	Fatal case report (forensic toxicology)	1	Ingestion of powdered *T. iboga* root containing 7.2% ibogaine; non-standardized ethnopharmacological preparation; quantified ibogaine and ibogamine in ingested material and postmortem biological samples by GC-MS/MS; co-ingestion of methadone and diazepam at therapeutic concentrations.	Death occurring approximately 12 h after ingestion of powdered iboga root; postmortem toxicological analyses demonstrated significant systemic exposure to ibogaine (blood concentrations up to 1.27 μg/mL) and ibogamine, with extremely high gastric concentrations (53.5 μg/mL); death attributed to iboga ingestion in the context of concomitant methadone and diazepam use, indicating a fatal toxicological interaction rather than isolated ibogaine exposure.	Single fatal case; polysubstance exposure confounds attribution of causality to ibogaine alone; non-standardized plant preparation with variable alkaloid content; absence of premortem ECG or clinical monitoring; limited generalizability despite robust analytical confirmation of exposure and cause-of-death attribution.
Papadodima et al. (2013) [49]	Fatal case report (forensic pathology and toxicology)	1	Ibogaine use in a non-medical, unregulated setting; formulation and exact administered dose not reported; postmortem blood concentration of ibogaine measured at 2.0 mg/L; no standardized pharmaceutical preparation; exposure confirmed by toxicological analysis.	Sudden death occurring approximately 12–24 h after ibogaine exposure; autopsy revealed advanced liver cirrhosis with severe fatty infiltration; death temporally associated with ibogaine use in the context of significant pre-existing hepatic pathology, raising concern for impaired metabolism and increased systemic exposure; cardiac arrhythmia considered a plausible mechanism given the known electrophysiological effect of ibogaine.	Single fatal case; absence of premortem ECG or cardiac monitoring; lack of precise dosing and formulation details; significant pre-existing liver disease represents a major confounder; causality cannot be attributed exclusively to ibogaine despite high blood concentration and close temporal relationship; limited generalizability.
Henstra et al. (2017) [46]	Case report with toxicokinetic analysis (LC-MS/MS)	1	Repeated oral ingestion of internet-purchased ibogaine capsules over approximately 12 h (total dose ≈ 1400 mg); non-regulated, non-medical setting; plasma ibogaine and noribogaine concentrations quantified by validated LC-MS/MS; maximum ibogaine concentration 1.45 mg/L; noribogaine peak concentration 0.569 mg/L with prolonged persistence.	Marked QTc prolongation (maximum QTc 647 ms) associated with multiple clinically significant cardiac arrhythmias, including atrial tachycardia, ventricular tachycardia, and torsades de pointes; QTc prolongation and arrhythmic risk persisted for up to 12 days after ingestion, extending well beyond clearance of parent ibogaine; toxicokinetic modelling demonstrated that prolonged cardiotoxic effects were temporally and quantitatively aligned with sustained noribogaine exposure rather than ibogaine itself, supporting a metabolite-driven mechanism of delayed cardiotoxicity.	Single-case design; non-standardized and unregulated ibogaine product; absence of CYP2D6 genotyping; potential contribution of transient hypokalemia and hypomagnesaemia as pro-arrhythmic cofactors; lack of controlled dosing conditions limits generalizability despite robust temporal, electrophysiological, and toxicokinetic evidence.
Kontrimavičiūtė et al. (2006) [51]	Forensic post-mortem toxicology study (case-based tissue distribution analysis)	1	Fatal ingestion of *T. iboga* root bark (non-standardized ethnopharmacological preparation); exact dose unknown; post-mortem quantification of ibogaine and noribogaine in blood, bile, and multiple tissues using validated LC–ESI–MS/MS.	Extensive systemic and tissue distribution of ibogaine and noribogaine was demonstrated post-mortem, with highest concentrations in spleen, liver, brain, and lung; both compounds shown to cross the blood–brain barrier and to be excreted via bile; high tissue-to-blood concentration ratios indicate marked lipophilicity and tissue sequestration, supporting prolonged biological persistence and mechanistic plausibility for delayed and systemic toxicity following iboga ingestion.	Single fatal case; absence of controlled dosing or timing data; polysubstance exposure not fully characterizable; post-mortem design precludes causal attribution of death mechanism but provides robust toxicokinetic and distributional evidence relevant to human safety risk.
Chèze et al. (2008) [50]	Forensic post-mortem case report with comprehensive toxicological and tissue distribution analysis	1	Fatal ingestion of *T. iboga* root (non-standardized ethnopharmacological preparation); recent exposure confirmed by LC–MS/MS detection of ibogaine and noribogaine in blood, urine, bile, gastric contents, liver, lungs, vitreous humor, spleen, and hair; no co-ingested licit or illicit drugs or alcohol detected.	Systemic detection of ibogaine and noribogaine across all post-mortem biological matrices, including incorporation into hair, consistent with recent high-level exposure; autopsy established drowning as the immediate cause of death, with a concomitant myocardial abnormality (myocardial bridging); the widespread presence of iboga alkaloids supports acute intoxication preceding death and underscores the potential for central nervous system impairment and loss of situational awareness contributing indirectly to fatal outcomes.	Single fatal case; death attributed primarily to drowning rather than a defined cardiotoxic event; absence of quantitative dose reconstruction; potential contribution of pre-existing myocardial abnormality; post-mortem design limits causal attribution while providing robust forensic confirmation of exposure and distribution.
Mestre et al. (2024) [47]	Clinical case report (life-threatening cardiotoxicity)	1	Oral ibogaine exposure, 200 mg, administered in an alternative detoxification setting; no co-ingestion of other QT-prolonging drugs; normal baseline electrolytes; no structural heart disease identified.	Acquired long-QT syndrome (QTc up to 636 ms) associated with polymorphic ventricular tachycardia (torsade de pointes) and multiple episodes of cardiac arrest shortly after exposure; recurrent malignant arrhythmias requiring repeated defibrillation and intensive care admission; gradual QTc normalization over 8 days following supportive management; findings demonstrate severe cardiotoxicity occurring at a low ibogaine dose, independent of electrolyte imbalance or underlying structural cardiac disease.	Single-case design; absence of pharmacokinetic measurements (ibogaine/noribogaine plasma levels not quantified); lack of genetic testing for congenital long-QT syndrome; exposure occurred in a non-regulated clinical context, limiting dose verification and generalizability despite strong temporal and clinical association.

**Table 3:** Preclinical Studies Investigating Ibogaine Toxicity and Mechanisms of Action.

Author (Year)	Study Type	N	Exposure/Dosing	Main Safety Findings	Methodological Limitations
Scallet et al. (1996) [53]	In vivo experimental neurotoxicology study with interspecies comparison (rat vs. mouse)	No specified	Acute ibogaine administration in rats and mice; doses ≥100 mg/kg (route consistent with contemporaneous preclinical protocols); neurohistological cerebellar tissue evaluation using argyrophilic staining, calbindin immunoreactivity (Purkinje neurons), and markers of astrocytosis and microgliosis.	Ibogaine induced marked cerebellar neurodegeneration in rats, characterized by selective Purkinje cell loss, argyrophilic degeneration, loss of calbindin immunoreactivity, astrocytosis, and microgliosis, predominantly affecting the cerebellar vermis. In contrast, no comparable neurodegenerative changes were observed in mice at equivalent exposures, demonstrating a clear species-specific vulnerability to ibogaine-induced cerebellar toxicity.	Acute exposure model with high doses relative to human use; lack of quantitative behavioural correlations; absence of pharmacokinetic measurements; interspecies differences limit direct translational inference but strongly support species-dependent neurotoxic risk.
Helsley et al. (1997) [54]	In vivo behavioural pharmacology study	N = 12	Ibogaine administered intraperitoneally: – Phase I: 50 mg/kg i.p., twice, ~8 h apart (dose previously associated with cerebellar neurotoxicity in rats); – Phase II: acute dose range administered 20 min before testing; – Phase III: 30 mg/kg i.p. administered immediately post-session across repeated days.	Ibogaine produced a dose-dependent reduction in response rate (psychomotor slowing) during maze performance, without impairing task acquisition, working memory, or task efficiency (% correct arm choices). At 30 mg/kg, ibogaine-treated rats committed fewer errors than vehicle-treated controls in a previously learned task. No evidence of learning or memory deficits was observed despite exposure to doses previously linked to cerebellar neurotoxicity in separate histopathological studies.	Behavioural endpoints only; absence of concurrent histopathological or neurochemical assessment; motor slowing may confound cognitive interpretation; intraperitoneal dosing and acute/subacute exposure limit translational relevance to human oral use.
Glick et al. (2000) [55]	Pre-clinical comparative mechanistic analysis integrating multiple rat experiments	Rat multiple cohorts	Ibogaine (~40 mg/kg, i.p.) versus 18-methoxycoronaridine (18-MC; ~40 mg/kg, i.p.); additional higher-dose toxicity assessments (≥100 mg/kg)	Both ibogaine and 18-MC reduce self-administration of opioids, cocaine, ethanol, and nicotine and attenuate opioid withdrawal signs. Effects are mediated by reduced reinforcing efficacy and modulation of mesolimbic dopamine signalling. Ibogaine uniquely increases extracellular serotonin and interacts with NMDA, sigma-2 receptors, sodium channels, and the serotonin transporter. Ibogaine produces dose-limiting adverse effects, including tremors, bradycardia, and cerebellar neurotoxicity at ≥100 mg/kg, whereas 18-MC retains antiaddictive efficacy with a substantially improved safety profile.	Heterogeneous experimental protocols; absence of standardized toxicological thresholds; non-unified sample sizes; findings limited to rodent models; translational relevance to humans remains inferential.
Xu et al. (2000) [52]	In vivo dose–response experimental study in rats (acute neurotoxicity assessment)	N = 6	Single intraperitoneal (i.p.) administration of ibogaine at 25, 50, 75, or 100 mg/kg; saline control. Histopathological assessment using silver staining (degenerating neurons), GFAP immunohistochemistry (astrocytosis), and calbindin immunolabeling (Purkinje cells).	Clear dose-dependent cerebellar neurotoxicity. Severe Purkinje cell degeneration and Bergmann astrocyte gliosis are observed in all animals at 75 and 100 mg/kg. Partial neurotoxicity at 50 mg/kg (2/6 rats affected), characterized mainly by astrocytosis. No histopathological abnormalities were detected at a dose of 25 mg/kg, establishing this dose as the no-observable-adverse-effect level (NOAEL) for acute cerebellar toxicity in rats.	Acute, single-dose design; intraperitoneal route not representative of human oral exposure; supratherapeutic dose range relative to human use; species-specific vulnerability of rat cerebellar Purkinje cells limits direct translational inference.

**Table 4:** Botanical sources of ibogaine and related iboga-type alkaloids, plant material used, and representative extraction approaches reported in the literature.

Plant Source (Species)	Plant Material Used	Geographic Origin	Preparation/Extraction Method (Summary)
*Tabernanthe iboga*	Root bark; minor amounts in leaves and seeds	Central Africa (Gabon, Congo, Cameron)	Classical maceration or percolation of powdered root bark with alcoholic or hydroalcoholic solvents, followed by successive acid–base extraction to obtain crude alkaloid fractions; isolation of ibogaine typically as hydrochloride salt [56,61,65,66]
*Tabernanthe iboga*	Root bark	Central Africa	Modern analytical profiling using LC-MS/MS and LC-HRMS/MS applied to aqueous or hydroalcoholic root extracts, revealing complex alkaloid and phenolic profiles beyond ibogaine [67]
*Voacanga africana*	Seeds and stem bark	West and Central Africa	Acid–base extraction of total alkaloids from seeds or bark; isolation of voacangine as a major constituent, subsequently used as a semisynthetic precursor for ibogaine production [61]
*Peschiera affinis*	Roots and stem	Brazil (Ceará)	Sequential cold extraction with hexane and ethanol, followed by conventional acid–base alkaloid extraction and chromatographic fractionation (silica gel, Sephadex LH-20) for isolation of individual iboga-type alkaloids [33]

## Data Availability

No new data were created or analyzed in this study. Data sharing is not applicable to this article.

## Abbreviations

18-MC	18-Methoxycoronaridine
AE	Adverse event
α	Level of statistical significance
AMPA	α-Amino-3-hydroxy-5-methyl-4-isoxazolepropionic acid receptor (AMPA)
BDNF	Brain-derived neurotrophic factor
cAMP	Cyclic adenosine monophosphate
CDT	Comissão de Dissuasão da Toxicodependência (Drug Addiction Dissuasion Commission)
CID-11	Classificação Estatística Internacional de Doenças e Problemas Relacionados com a Saúde, 11.ª edição (International Classification of Diseases, 11th Revision—ICD-11)
CoA	Certificate of Analysis
CREB	cAMP response element-binding protein
DA	Dopamine
DAT	Dopamine transporter
DMN	Default mode network
D2	Dopamine D2 receptor
DSM-5	Diagnostic and Statistical Manual of Mental Disorders
EAG	Serious adverse event
ECG	Electrocardiogram
EMA	European Medicines Agency
EMCDDA	European Monitoring Centre for Drugs and Drug Addiction
ENLCD	Estratégia Nacional de Luta Contra a Droga (National Drug Strategy)
FDA	Food and Drug Administration
FFUC	Faculdade de Farmácia da Universidade de Coimbra (Faculty of Pharmacy, University of Coimbra)
GABA	Gamma-aminobutyric acid
GABA-A	Gamma-aminobutyric acid receptor A
GCP	Good Clinical Practice
GDNF	Glial cell line-derived neurotrophic factor
GITA	Global Ibogaine Therapy Alliance
HCl	Ibogaine hydrochloride
ICH	International Council for Harmonisation
IPN	Interpeduncular nucleus
ISRS	Inibidores seletivos da recaptação da serotonina (Selective serotonin reuptake inhibitors—SSRIs)
ITT	Intention-To-Treat
Ki	Inhibition constant
KOR	κ-Opioid receptor
MAPK/ERK	Mitogen-activated protein kinase/Extracellular signal-regulated kinase
MHb	Medial habenula
MOR	µ-Opioid receptor
mTOR	Mammalian target of rapamycin (Alvo da rapamicina em mamíferos)
NAc	Nucleus accumbens
nAChR	Nicotinic acetylcholine receptor
NMDA	N-Methyl-D-aspartate receptor
NSP	Novas substâncias psicoativas (New psychoactive substances—NPS)
OMS	Organização Mundial da Saúde (World Health Organization—WHO)
PFC	Prefrontal cortex
PI3K/Akt	Phosphatidylinositol 3-kinase/Protein kinase B
PLCγ	Phospholipase C gamma
PPS	Per-Protocol Set
PTA	Purified total alkaloid extract
RGPD	Regulamento Geral de Proteção de Dados (General Data Protection Regulation—EU 2016/679)
RRMD	Redução de riscos e minimização de danos (Risk Reduction and Harm Minimization)
SERT	Serotonin transporter
SNc	Substantia nigra pars compacta
SS	Safety Set
STROBE	Strengthening the Reporting of Observational Studies in Epidemiology
SUD	Substance Use Disorder
TA	Total alkaloid extract
TUS	Transtorno por Uso de Substâncias (Substance Use Disorder)
UNODC	United Nations Office on Drugs and Crime
VTA	Ventral tegmental area
WDR	World Drug Report
WHO	World Health Organization
